# Avoiding obstacles while intercepting a moving target: a miniature fly's solution

**DOI:** 10.1242/jeb.243568

**Published:** 2022-02-15

**Authors:** Samuel T. Fabian, Mary E. Sumner, Trevor J. Wardill, Paloma T. Gonzalez-Bellido

**Affiliations:** 1 Department of Physiology, Development, and Neuroscience, University of Cambridge, Cambridge CB2 3EG, UK; 2 Department of Bioengineering, Imperial College London, London SW7 2AZ, UK; 3 Department of Ecology, Evolution and Behaviour, University of Minnesota, Saint Paul, MN 55108, USA

**Keywords:** Insect, Flight, Navigation, Predation

## Abstract

The miniature robber fly *Holcocephala fusca* intercepts its targets with behaviour that is approximated by the proportional navigation guidance law. During predatory trials, we challenged the interception of *H. fusca* performance by placing a large object in its potential flight path. In response, *H. fusca* deviated from the path predicted by pure proportional navigation, but in many cases still eventually contacted the target. We show that such flight deviations can be explained as the output of two competing navigational systems: pure-proportional navigation and a simple obstacle avoidance algorithm. Obstacle avoidance by *H. fusca* is here described by a simple feedback loop that uses the visual expansion of the approaching obstacle to mediate the magnitude of the turning-away response. We name the integration of this steering law with proportional navigation ‘combined guidance’. The results demonstrate that predatory intent does not operate a monopoly on the fly's steering when attacking a target, and that simple guidance combinations can explain obstacle avoidance during interceptive tasks.

## Abstract (precis)

**Highlighted Article:** Small predatory flies can simultaneously combine information about a moving target and a nearby obstacle to safely navigate to their goal. These flies sum the steering requirements of each task and take an intermediate path, much as humans do.

## INTRODUCTION

Navigating through cluttered environments filled with static or moving obstacles is a daily occurrence for humans and other animals, yet remains major challenge for modern robotics (Masehian and Sedighizadeh, 2007; Raja and Pugazhenthi, 2012). Many robotic systems use reflections of actively emitted soundwaves (Borenstein and Koren, 1989) or lasers (An and Wang, 2004) to detect surfaces and provide a distance map to their surroundings. Such information can then be used to form a path-plan of how to traverse the environment without making contact with objects whilst navigating to a goal (Gilbert and Johnson, 1985; Raja and Pugazhenthi, 2012). Using first-order (time-derived) visual information is energetically cheaper than 3D reconstruction (Raviv and Joarder, 2000) or formal path planning. The cost effectiveness and reduced sensor requirements of simple visual cues may be why they appear widely used by animals during localised navigation, pursuit or interception (Brighton et al., 2017; Collett and Land, 1975; Fabian et al., 2018; Ghose et al., 2006; Haselsteiner et al., 2014; Land, 1993; Land and Collett, 1974; Trischler et al., 2010; Warren et al., 2001). Reactive methods do not require absolute maps or knowledge of exact target location, speed, etc. Instead, they heuristically react to salient stimuli. Indeed, biologically inspired and vision-based obstacle avoidance has been applied to robots and computer vision frameworks, using the system described in flying honeybees (Srinivasan et al., 1996), i.e. by balancing the surrounding optic flow whilst navigating effective corridors (Santos-Victor et al., 1993; Souhila and Karim, 2007; Srinivasan et al., 1999). In turn, the building of robotic systems informs our understanding of biological navigation (Bagheri et al., 2017; Expert and Ruffier, 2015; Franceschini et al., 2007).

**Fig. 1.**
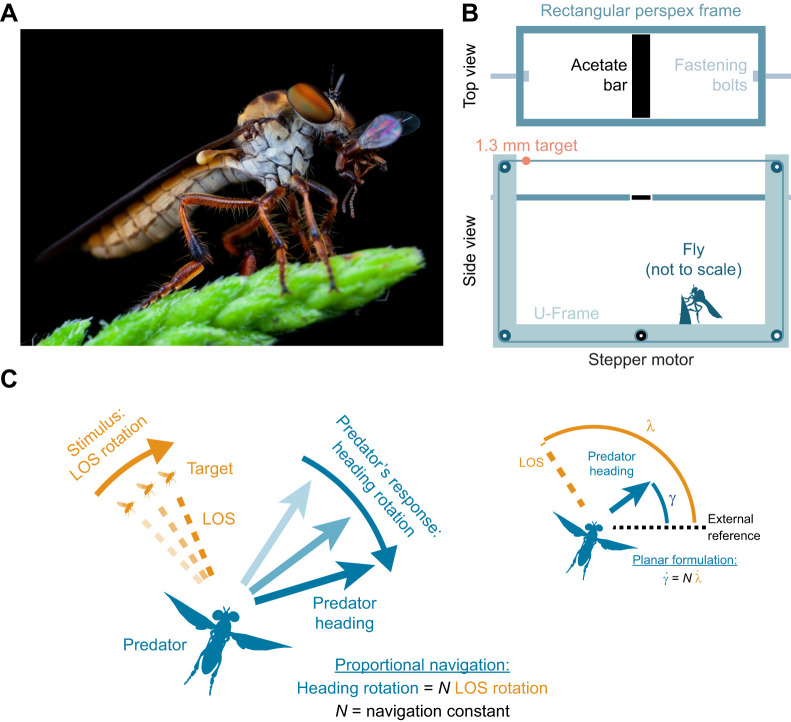
**Research species, apparatus, and the proportional navigation guidance law.** (A) *Holcocephala fusca* feeding on prey. (B) The obstacle presentation apparatus. The obstacle is a black acetate bar placed on a rectangular Perspex frame (top). The frame was placed horizontally on the arms of a U-frame (bottom). A loop of fishing line was guided by the pulleys at the corners of the U-frame. A 1.3 mm bead looped onto the fishing line simulates a prey item. The movement of this fishing line loop was controlled via a stepper motor at the base of the U-frame. (C) Left: the principle of proportional navigation is demonstrated figuratively. Rotation of the line-of-sight (LOS) is magnified by a navigation constant (*N*) and applied the predator's heading. Right: the elements of the geometry of proportional navigation (pro-nav) are described, demonstrating how both the change in the LOS angle (λ) and of the heading angle (γ) are taken from a common external reference frame.

Fitting navigational behavioural algorithms is becoming an increasingly popular method for determining the logic of fast reactive behaviour such as obstacle avoidance and target pursuit (for review, see Hein et al., 2020). The ability to navigate through cluttered environments and avoid obstacles has been tested in many animals, including locusts (Robertson and Johnson, 1993), fruit flies (van Breugel and Dickinson, 2012), pigeons (Lin et al., 2014) and humans (Fajen et al., 2003; Warren et al., 2001). However, in these instances, obstacle avoidance was the only goal. Navigating around an obstacle is more challenging when a particular location acts as a target because the aversion to obstacles must be balanced by the navigational goal. This task has been studied in humans with behavioural dynamics, where the participant navigated to a designated target on the floor around potential obstacles (Fajen and Warren, 2003). When navigating toward a static goal, humans incorporate optic flow cues from the surrounding environment with target-centred steering in a linear combination (Warren et al., 2001), demonstrating how multiple guidance requirements can be incorporated. The obstacle avoidance problem is further complicated when the target is in motion, or when obstacles temporarily obscure the target. Even extremely small animals often deal with this problem on a near continuous basis as they traverse the world, yet we have little empirical knowledge of the guidance rules that enable them to do so. For example, the predatory robber fly *Holcocephala fusca* is minute (6 mm body length) and hunts even smaller aerial invertebrates from a perch (Fig. 1A). *Holcocephala fusca* generally perches with a clear view of the sky, but errant branches and obstacles may still obscure some of the area above the fly. In such cases the fly must steer, both to intercept the target and to avoid the obstacle. The demanded steering of these tasks may at times conflict, requiring one response to be prioritised above the other. *Holcocephala fusca* further presents an excellent model in which to study obstacle avoidance because (1) its predatory interception mechanism is predictable and well described (Fabian et al., 2018) and (2) its small size and fast behaviour (predatory flights are <1 s long) require rapid reactions with minimum computational effort. The steering behaviour by which *H. fusca* navigates to its prey (Fabian et al., 2018) has been shown to be analogous to that of falcons (Brighton et al., 2017), hawks (Brighton and Taylor, 2019) and modern guided missiles (Shneydor, 1998). The system used is an analogue of proportional navigation (pro-nav), in which the rotation of the line-of-sight to the target (LOS) is magnified and applied to the rotation of the velocity of the interceptor. Eqn 1 represents pure proportional navigation:
(1)

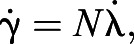
where 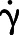 is the rotation in the heading of the interceptor, 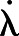 is the rotation of the LOS relative to the external world and *N* is the navigation constant. The navigation constant provides the gain for the reactive system, and therefore the path taken by the interceptor depends on it. Correctly setting the value of *N*, the gain of the interceptor, is critical to using pro-nav to successfully explain an interception behaviour. The predatory behaviour of *H. fusca* is best described by a navigational constant of *N*≈3. This is similar to that found in falcons (Brighton et al., 2017) and engineered systems (Shneydor, 1998), while hawks and other predatory flies operate at a lower constant value (Brighton and Taylor, 2019; Fabian et al., 2018).

Here, using *H. fusca* as a model, we address two fundamental questions about goal-directed navigation in the presence of obstacles: (1) how is an interceptive path altered when an obstacle challenges its success and (2) how does the guidance system respond when the obstacle obscures the target? To answer both of these questions, we tested the ability of *H. fusca* to navigate to a moving artificial target under field conditions in the presence of a single obstacle, which in some trials also temporarily obscured the target. We found that the rate of visual expansion of the obstacle explains how the interceptive behaviour of *H. fusca* changes in the presence of an obstacle. Therefore, at certain times, the angular expansion of the obstacle on the retina takes priority, but this information is constantly integrated with the guidance commands required to intercept the target. When the target was briefly occluded by the obstacle, *H. fusca* completed the interception, but the task was abandoned if visual contact was lost for more than approximately 70 ms.

## MATERIALS AND METHODS

### Obstacle and target presentation

*Holcocephala fusca* (Bromley 1951) was presented with a moving target (black bead, 1.3 mm diameter) affixed to a loop of fishing line stretched around a Perspex U-frame and moved via pulleys and a stepper motor (see Wardill et al., 2015) at 0.32 m s^−1^. The moving stimulator, ‘fly teaser’ frame also held the obstacle: a bar of black acetate, placed just below the path of the target (Fig. 1B). The bar had two alternative widths: thin (2.5 cm) or thick (5 cm), equating to subtended angle of 4.8 or 9.5 deg, respectively, when placed at 30 cm from a perched animal prior to take-off. The exact placement of the bar and the initial trajectory of the fly determined whether the object became an obstacle in the flight path and whether it obscured the target.

All flight recordings were captured under field conditions. As *H. fusca* remained on their perch, the U-frame and hanging obstacle (Fig. 1B) were placed overhead by hand. The process of manoeuvring the equipment overhead caused the majority of flies to take off (exact statistics unrecorded); the flights within this paper feature those flies that remained perched as the apparatus was set up around them. The obstacle was created by painting an acetate sheet with black acrylic paint (Crawford & Black Paints), to the point where light could not visibly be seen through it. This acetate bar was stretched taught across a rectangular Perspex frame shown in Fig. 1B. The rectangular frame was held below the path of the target in the U-frame by means of M6 bolts that allowed it to rotate along its longitudinal axis, keeping the rectangular tray horizontal even if the U-frame were held at an angle to the ground.

Targets took the form of a 1.3 mm silver coloured reflective bead tied onto clear fishing line (Berkley Trilene; 2 lb breaking strength). All targets were moved by means of a stepper motor at a constant speed of 0.32 m s^−1^ (reconstructed s.d. ±0.02 m s^−1^). The direction of travel was consistently held as parallel to the ground and aligning with the head-to-tail axis of the fly (i.e. the target was vertically above but coming towards them frontally in all trials).

### Capturing and digitising trajectories

Two synchronised Photron Fastcam SA2 cameras with overlapping fields of view were used to film flight behaviours at 1000 frames s^−1^. After a behaviour was recorded, the two cameras were calibrated by moving a known-sized checkerboard through the behaviour space in the view of both cameras. The position of the fly, target and obstacle corners were then digitised by hand through successive frames of both camera views and positions converted into *xyz* Cartesian coordinates (using custom-written MATLAB scripts; Wardill et al., 2017). Trajectories were then smoothed to account for noise generated in the tracking process using a custom written script that penalised smoothed trajectories based on their fit to the raw data and their jerk (third-order derivative of position) (Dey and Krishnaprasad, 2012).

### Modelling and simulations

All modelling and analyses were conducted in MATLAB 2018a. Simulations took the form of sequential, discrete-interval time steps in which the time-delayed inputs were used to derive the predicted steering of the simulated fly. The linear speed of the fly and model were matched by scaling the simulated fly's velocity in accordance with the true fly's linear acceleration.

Flight simulations were run and evaluated using a sweep through potential constants (each given an envelope and step size). The constant combinations for each sweep were as follows: pro-nav constant *N* (2 to 6 in increments of 0.1), pro-nav time delay (*T*_dpn_; 15 to 45 ms in increments of 1 ms), obstacle aversive constant *c* (0.00 to 0.70 in increments of 0.01), obstacle aversive time delay (*T*_doa_) between rate of object expansion and turning response (30 to 130 ms in increments of 10 ms) and field-of-view (FOV; 40 to 180 deg in increments of 10 deg). These envelopes were set based on preliminary observations of the model behaviour. The FOV was centrally defined by the LOS, assuming that the visual axis of the fly moved with the target. The assumption that the fly fixates on the target was based on the finding that *H. fusca* looks at the target and tracks it while moving its head before take-off, and that the eye has a centralised region of remarkably acute vision (for a compound eye) (Wardill et al., 2017). Flight simulation used discrete time interval steps of 1 ms, to match the temporal resolution of the recorded data. Model performance was scored by the percentage of flight time in which the distance between the simulated *H. fusca* and real fly was shorter than 5% of the distance that they had flown (e.g. if they had flown 10 cm, they needed to be within 5 mm), in keeping with similar metrics in published literature (Brighton et al., 2017).

## RESULTS

From stereo high-speed videography, we digitally reconstructed 26 flights of *H. fusca* taking off after the moving target in the presence of an obstacle. In 17 of the recorded trajectories, the target was temporarily obscured from the fly by the obstacle. If the target was obscured by a thin bar, *H. fusca* terminated the attack 14% of the time (1/7 trials). In contrast, 100% of the trials (10/10) were terminated when the thick bar acted as the occluder. In addition, in the presence of the thick bar, one flight was terminated without the target having been occluded. The mean (all means are presented ±s.e.m. unless otherwise indicated) duration of target obscurement was higher for terminated trajectories (164±23 ms, *n*=11) than for completed trajectories (65±5 ms, *n*=6) (*t*=3.1, *P*<0.01). The minimum duration of target obscurement that resulted in terminated trajectories was 60 ms (thin bar), and the maximum 306 ms (thick bar). For completed trajectories (14 in total), the minimum duration of target obscurement was 60 ms and the maximum was 72 ms (thin bar). Thus, there was a correlation between the duration of obscurement and the likelihood that the attack continued thereafter, such that trajectories with brief obscurements were more likely to be continued (re-engaged after the obscurement) (Wilcoxon rank sum, *Z*=2.7, *P*=0.007). The bar also took up a smaller angular size in the FOV of the fly during target obscurement in re-engaged trajectories (mean±s.d. 6±3 deg) than in terminated trajectories (29±21 deg) (*t*=69.6, *P*<0.001). However, these differences were largely accounted for by the difference in the angular size of the thin and thick bars during the first half of the trajectories (8±3 and 27±9 deg, respectively).

### Pro-nav in the presence of obstacles and occluders

**Fig. 2.**
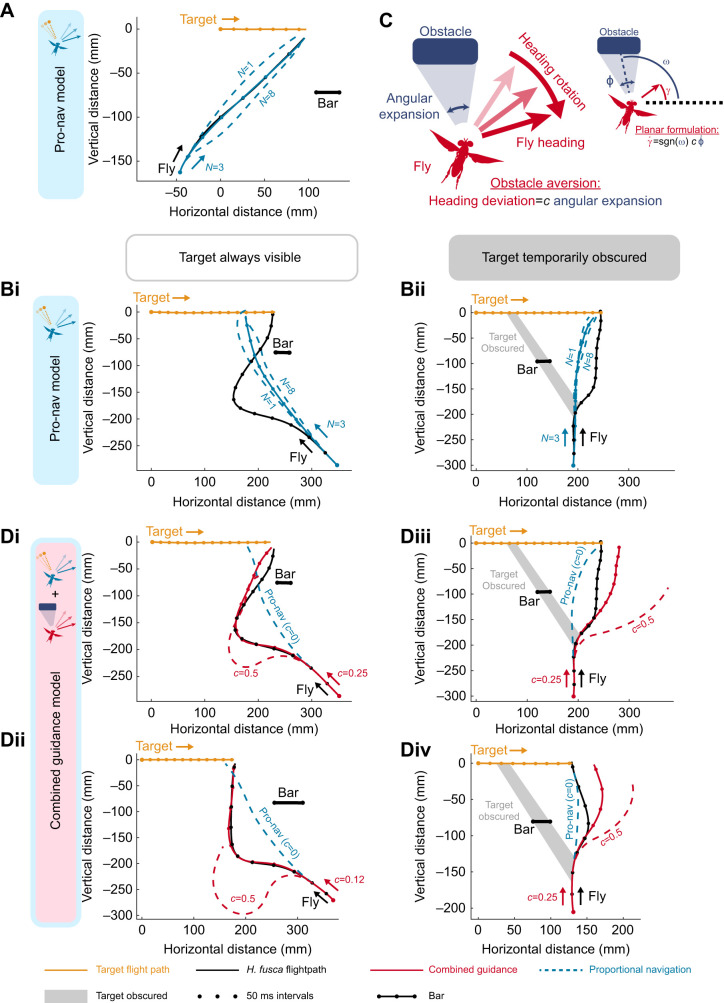
**Guidance simulations and real *H. fusca* interceptive paths in the presence of an obstacle.** (A) *Holcocephala fusca* intercepts the moving target in the presence of a 2.5 cm obstacle. A simulation of pro-nav, moving at the same speed as the fly, is depicted in blue (dashed lines at navigational constant values of *N*=1 and *N*=8, solid line at *N*=3, score=100%, dots mark 50 ms intervals in all panels). (B) Trajectories of *H. fusca* intercepting a moving target that is (i) always visible and (ii) temporarily occluded by a 2.5 cm width obstacle are simulated using a proportional navigation steering model [dashed lines at *N*=1 and *N*=8, solid line at *N*=3, time delay (pro-nav) *T*_dpn_=28 ms, scores=(i) 19% and (ii) 56%]. (C) Left: the principles underlying the obstacle aversion model. Right: the geometry underlying the obstacle aversive element of the new model. ω is the angle from the LOS to the obstacle and the velocity of the predator. φ is the angular size of the obstacle, from which the time derivative (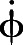) is input into the control law. (D) Combined guidance simulations are fitted to trajectories in which the target was (i,ii) always visible or (iii,iv) temporarily obscured by the obstacle (red line). Grey shaded area represents when the target was obscured by the obstacle. Simulations are shown for pro-nav (*c*=0), the individual best-fitting value for *c*, and a high value of *c* (*c*=0.5). Fitted gains and scores were as follows: (i) score=80%, *N*=3.8, *T*_dpn_=25 ms, *c*=0.25, time delay (obstacle-avoidance) *T*_doa_=90 ms, FOV=120 deg; (ii) score=100%, *N*=3.6, *T*_dpn_=36 ms, *c*=0.12, *T*_doa_=80 ms, FOV=100 deg; (iii) score=55%, *N*=3.2, *T_dpn_*=30 ms, *c*=0.15, *T*_doa_=90 ms, FOV=140 deg; (iv) score=65%, *N*=4.3, *T*_dpn_=28 ms, *c*=0.36, *T*_doa_=90 ms, FOV=100 deg.

As expected, when the bar's location did not interfere with flight course of the attack, pro-nav explained the interceptive trajectory (Fig. 2A). In contrast, if *H. fusca* flew in close proximity to the bar, and based on the current trajectory of the target the bar could act as a visually salient obstacle, *H. fusca* deviated its pathway dramatically (*n*=8). Pro-nav was clearly not successful at predicting the predatory flight's path in such conditions (example in Fig. 2Bi). The flies also deviated their flight path away from the course predicted by the pro-nav model when the bar temporarily obscured the target (*n*=6) (example shown in Fig. 2Bii).

### The combined guidance model

The course *H. fusca* took to the target in the presence of a salient obstacle invariably took it further away from the obstacle. This suggested the inclusion of an additional, obstacle-aversive aspect to the navigational system. This aversive aspect must use information that is readily available to the flies (Fig. 2C). We assume that the fly did not have knowledge of the physical location or absolute distance to the obstacle. Under such conditions, the rate of change in the angular size of the obstacle (resulting from the translation of *H. fusca* during flight) becomes a crucial cue. This is because a visual expansion on the retina is indicative of both the proximity and approach speed of an obstacle. Thus, greater rates of expansion of objects should generate larger aversive turning behaviours. From a combination of the rate of change in angular size, and the heading angle of the target, a very simple avoidance algorithm can be generated, given by Eqn 2:
(2)

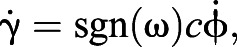
where 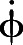 is the rate of change in the angular width of the obstacle and ω is the bearing of the obstacle from the navigator's heading, whose sign (sgn) denotes the direction of the angle (to the left or to the right of the interceptor). *c* is a dimensionless constant. For this model to work, it is important to consider that obstacles that are receding and reducing in angular size are unlikely to be of relevance to collision, thus it is assumed the aversive element should only be active when 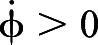. Furthermore, the navigator is likely to be visually fixated on the target, especially concerning times when the target is moving, as in predation. This may result in a FOV limitation around the target, or a region of interest. This limitation may require that only obstacles within a particular angular range from the LOS to the target are of interest. This can be added to the pure pro-nav equation to generate a model that will both intercept a moving target and avoid physical obstacles. We name this model ‘combined guidance’ as it is a linear combination of the control requirements, as also found in humans (Warren et al., 2001). This ‘combined guidance’ model is shown by Eqn 3:
(3)

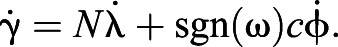
The supposition that the obstacle avoidance operates within a limited FOV is affirmed by flight trajectories that agreed with pro-nav despite taking them within 15 mm of hitting the obstacle, when the LOS to the target faces away from the obstacle (Fig. S1).

### Flight simulations

When the bar was presented in close proximity to the fly, pro-nav alone was a poor fit for the completed trajectories (example shown in Fig. 2B; full data set in Fig. S2) (i.e. those in which the fly successfully intercepted the target; mean score=39±7%, *n*=14). The addition of expansion avoidance improves the fit of the model greatly (mean score=79±4%, *n*=14; examples shown in Fig. 2D). On average, the best fits were *c*=0.22±0.02 for the avoidance constant, 86±9 ms for the obstacle avoidance time delay and 86±2 deg for the mean FOV. With regards to the pro-nav component, the average best fit was 3.6±0.2 for *N* and 30±2 ms for the delay. Both pro-nav *N* and time-delay values are consistent with previous findings (Fabian et al., 2018).

For salient objects, the obstacle avoidance model performance was always an improvement over pure pro-nav (examples shown in Fig. 2D; full data set in Fig. S2). The best fit for the obstacle avoidance model corresponded to the trials in which the target was never obscured by the obstacle, with the mean performance score=86±11%, *n*=7 (example shown in Fig. 2Di,ii). In contrast, for flights with obscured targets, the mean performance score=73±6%, *n*=7 (example shown in Fig. 2Diii,iv). Interestingly, the flight trajectory produced by the combined model appears a good fit when the target is initially obscured. However, after the target emerges on the other side of the obstacle, the flies make a turn much sharper than predicted by the combined guidance model (examples shown in Fig. 2Diii,iv).

### Alternative re-engagement turn hypotheses

If an attack continued after the target had been obscured, this was termed re-engagement. From the current model of *H. fusca* guidance, there are two evident means by which a sharper turn back towards the target during re-engagement could be engendered once the target becomes visible on the other side of the obstacle. The first is in the pro-nav unitless navigation gain constant *N*. Using a higher navigational constant in the combined guidance model (higher *N* value in Eqn 3) would allow for a faster turn back onto the interception course for a given LOS rotation rate (or potentially an effective saccadic turn onto an approximate collision course). A second explanation lies in the logical extension of the obstacle aversive algorithm, that of the optomotor inversion as detailed in *Drosophila* (Tammero et al., 2004). The initial equation's evasive actions of the combined guidance model were restricted to a target that increased in angular size (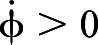 in Eqn 3). Removing this limitation results in the navigator being attracted to receding objects as well, which may explain the rapid turn back towards the target once the fly passes the obstacle (see Fig. S2).

We tested these two explanations independently; simulations of the combined model (Eqn 3) were run either with a much greater envelope values of the gain constant *N*, or receding obstacles were permitted to attract the fly's steering (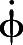 could take negative and positive values).

### High navigational constant (*N*) explanation

**Fig. 3.**
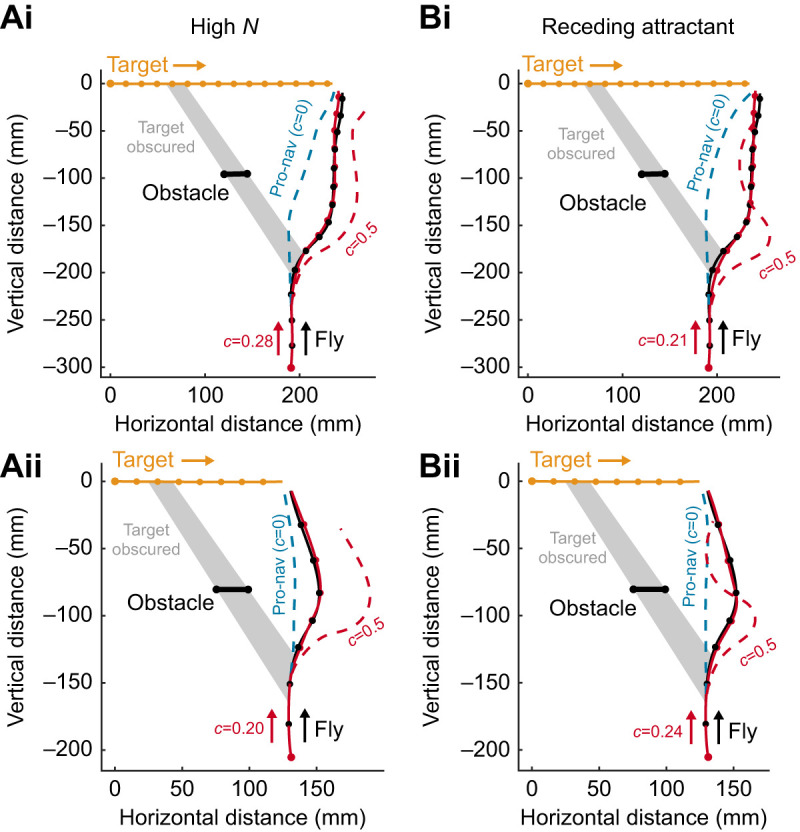
**Alternative explanations for target re-engagement alongside real *H. fusca* interceptive paths.** (A) Combined guidance simulations are fitted with a high-*N* envelope (7<*N*<15) (red line, dots mark 50 ms intervals). Grey shaded area represents when the target was obscured by the obstacle. The best-fitting gains are as follows: (i) score=100%, *N*=10, *T*_dpn_=28 ms, *c*=0.28, *T*_doa_=60 ms, FOV=60 deg; (ii) score=100%, *N*=11, *T*_dpn_=26 ms, *c*=0.20, *T*_doa_=70 ms, FOV=70 deg. (B) Combined guidance simulations are fitted without the obstacle deviation requirement (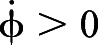), making obstacles reducing in angular size attractive to the fly's steering (red line, dots mark 50 ms intervals). Grey shaded area represents when the target was obscured by the obstacle. The best-fitting gains are as follows: (i) score=100%, *N*=3.8, *T*_dpn_=35 ms, *c*=0.21, *T*_doa_=70 ms, FOV=70 deg; (ii) score=100%, *N*=3.4, *T*_dpn_=30 ms, *c*=0.24, *T*_doa_=70 ms, FOV=70 deg.

Increasing the bounds of *N* of the combined model simulations improved the fit against the real trajectories in which the fly steered toward the target after it had been obscured by the obstacle (Fig. 3A). The best fitting gain was variable but in the region of *N*≈10 (mean *N*=10.1±2.2, *n*=7; examples shown in Fig. 3A). With this gain, the model score was improved for all re-engagement flights, but this effect was not significant (from mean score=73±6%, *n*=7, to mean score=80±6%, *n*=7, *t*=−1.17, *P*=0.28) Moreover, while the high navigation gain (*N*>7) combined guidance model effectively manages a re-curve that matches that of the fly, higher navigational constants overall significantly reduced the model fit for trajectories in which the target was not obscured (from mean score=86±11%, *n*=7, to mean score=69±7%, *n*=7, *t*=2.67, *P*=0.03).

### Receding attractant explanation

For the receding attractant model, the fitted *c*, a dimensionless constant in the combined guidance model (see Eqn 3), was 0.23±0.04 and the mean fitted avoidance delay was 87±2 ms. These values are similar to those of the initial combined guidance model. The receding-attraction model also significantly improved the fit of the navigational model to the path of the flies (Fig. 3B), when compared with the pure pro-nav model for all flights in which the target was successfully intercepted (from mean score 79±6%, *n*=14, to mean score 99±1%, *n*=14, *t*=−3.08, *P*=0.008). Importantly, this method maintains a similar navigation gain constant to the extant work on *H. fusca* (*N*≈3), and the performance of the simulation does not depend on whether the target was temporarily occluded or always visible (see Figs S3 and S4 for all 14 completed trajectories, fitted with the receding attract combined guidance model). The sensitivity of this best-fitting model's explanatory power to variation in each of the constants is demonstrated in Fig. S5.

## DISCUSSION

*Holcocephala fusca* are capable of avoiding static obstacles as they intercept moving targets. The combined guidance model (Eqn 3) demonstrates that obstacle avoidance can be the product of simple feedback laws that do not require absolute knowledge of distance, size or velocity (Fig. 3). The heuristic nature of the explanatory model proposed here for obstacle avoidance matches previous results that a simple feedback controller was sufficient to explain the interception trajectory of this *Holcocephala* species (Fabian et al., 2018). Pro-nav uses rotation of the target LOS to steer into a collision course with the target, instead of calculating or predicting the correct course based on absolute knowledge of target distance, speed, and heading. It is therefore not surprising that the obstacle aversion of *H. fusca* can be explained using approximate indexing of obstacle collision based on visual expansion rather than absolute knowledge of the distance to the obstacle. Our firm conclusions are limited by the relatively small number of trials that could be conducted (owing to limitations of working with live animals in the field). Collecting further trials, with the animal deviating because of obstacles with a greater range of widths, and added motion would allow for more concrete assertions. Importantly, their small size and manoeuvrability, on par with that of their prey, may allow *H. fusca* to rely fully on feedback guidance for interception. Larger predatory aerial animals may operate under more complex guidance. For example, bats employ predictive models for target tracking (Salles et al., 2021), and dragonflies are thought to perform interceptive path planning (Mischiati et al., 2015). In both cases, reactive systems are still needed to update the course owing to target accelerations. Our view is that the addition of an internal model of the target provides a solution for predators with reduced manoeuvrability aiming to catch smaller agile prey. An increase reliance on prediction of the future target's location may therefore be expected in those predators with relatively large (1) body size compared to prey, (2) sensorimotor delays and (3) constrained manoeuvrability. However, internal models of the external world are computationally expensive and, if erroneous, their output predictions may lead to disastrous task failure. By relying heavily on reactive strategies, predators with high manoeuvrability and speed of neural responses can avoid such drawbacks. Because *H. fusca* is a miniature robber fly, we expect its neural and mechanical delays to be small, allowing for successful captures with reliance on primarily reactive strategies.

Expanding stimuli are well known to generate strong behavioural responses in many species (Schiff et al., 1962), and represent one key characteristic of optic flow required to compute relative motion of the surroundings (Land, 1999; Schuster et al., 2002). Retinal expansion of objects also underpins escape responses in many species (Ache et al., 2019; Peek and Card, 2016; Robertson and Johnson, 1993), as well as mediating landing or avoidance responses in fruit flies (van Breugel and Dickinson, 2012). Although in this paper, expansion is discussed with response to a single distinct object, it is not necessarily treated as such in the animal’s neural pathways, as this would require continuous feature correspondence over time (Fermüller and Aloimonos, 1993). Instead, obstacle avoidance could be generated by more standard optomotor pathways, such as those already described in flying insects navigating around their environment (Krapp and Hengstenberg, 1996; Land, 1999; Lee, 1980; Pix et al., 2000; Schuster et al., 2002; Srinivasan et al., 1996; Tammero and Dickinson, 2002), implemented in modern robotics (Raviv and Joarder, 2000; Serres and Ruffier, 2017; Srinivasan et al., 1999), and which guide our own navigation of the world (Warren et al., 2001). In our setup, the obstacle is orders of magnitude closer to the animal than other objects in a similar FOV (i.e. treetops and distant foliage), and thus likely dominates the optic flow field during these short, upward-facing predatory attacks. Were the animal flying in a cluttered environment, a different representation of *H. fusca*’s obstacle avoidance element of combined guidance would likely be necessary; one that accounted for the entire optic flow field and summarised it for contribution to the pro-nav steering requirements similar to the model proposed for human navigation toward a static goal (Fajen et al., 2003; Warren et al., 2001). Future work would also benefit from testing other elements of the optic flow field during aerial interception, such as translational flow (Srinivasan and Zhang, 2004; Srinivasan et al., 1996), or whether expansion stimuli are unique in influencing the path of the fly. Rotational flow fields have been shown to influence conspecific tracking in hoverflies (Collett, 1980; Collett and Land, 1975), but in blowfly pursuit the optomotor system appears suppressed (Trischler et al., 2010). These results, along with our own, suggest that there may be diverse strategies in how wide-field optic flow is used during chasing behaviours.

As an explanation of the sharp turns of re-engaged trajectories, we would suggest that our second interpretation (that *H. fusca* navigate towards receding objects) is more probable. A sustained high navigation constant is liable to be unstable in real-world implementation (Fabian et al., 2018; Shneydor, 1998) and thus is unlikely. We have not directly modelled a mid-flight saccadic turn but have approximated it in our high-*N* obscurement simulations. It is beyond the scope of this work to assess a proposed mechanism for the effective ‘resetting’ of the course through a sharp turn onto an estimated interception course. Instead, to identify concretely whether contracting targets act as attractants, experimentation is required in which the width of an obstacle can be varied during the flight. In this regard, it must be noted that the receding attractant hypothesis is supported by its resemblance to the optomotor inversion toward expansion stimuli in the fruit fly *Drosophila melanogaster*. *Drosophila melanogaster* steering behaviour shows a bimodal response to expansion based on the polarity and visual speed, with strongly expanding objects creating aversive turns, whilst weakly expanding or contracting objects are actively steered towards (Reiser and Dickinson, 2013; Tammero et al., 2004). Although in the final formulation our combined guidance model is attracted to contracting objects and avoids expanding objects, further testing may illuminate whether, as in *D. melanogaster*, the optomotor inversion includes obstacles that are only weakly expanding.

We have demonstrated in this work that the steering of *H. fusca* during predation is not monopolised by pro-nav, and suggest that like humans, steering by this small fly results from the linear combination of multiple control systems (Warren et al., 2001). Although significant depth of analysis has been published for the tuning of the pro-nav controller (Fabian et al., 2018; Ho et al., 1965; Shneydor, 1998), the weighting optimality of the expansion avoidance element is less evident. Tuning *c* (combined guidance model; Eqn 3) to a greater value will steer the interceptor further from obstacles, but also cost more in terms of time and distance, potentially reducing the fly's ability to intercept targets. A time delay of 85 ms for the obstacle avoidance response is long compared with similar behavioural responses trigged by objects in fruit flies (which are closer to 50 ms; Fry et al., 2003; Tammero and Dickinson, 2002), and much longer than the best fit for the delay used in *H. fusca* pro-nav guidance (approximately 28 ms) (Fabian et al., 2018). This slow response could reflect the greater required integration time for the slower expansion signals (created by the greater distance between *H. fusca* and the obstacle than in comparable studies), or potentially the sharing of bandwidth with the pro-nav steering requirements. To discern further why this response delay is long, it would be advantageous to gather free-flight trajectories of *H. fusca* avoiding obstacles whilst not also tracking a target.

There are many additional questions about the combined guidance algorithm of *H. fusca* that remain unanswered. One such question is which metric of the target presents the best analogue of the fly's sense of the threat of collision. In this paper, we have used the most reduced information required, angular expansion, but this information could be extrapolated to infer the minimum time to contact (Subbarao, 1990) based on a principle termed tau theory (Lee et al., 1991, 1992). Although the generality of tau theory for describing the movement of animals in 3D environments has been questioned owing to its lack of consideration of accelerations and spatial dimensions of the observer (Tresilian, 1999), the principles could still be implemented in the obstacle avoidance of *H. fusca*.

How exactly the two guidance systems, potentially operating at different time delays (∼30 and ∼90 ms for pro-nav and obstacle avoidance, respectively), are integrated to form a response is uncertain but worthy of interest. There are many layers at which the two could interfere. Two major alternatives are as follows: (1) both pro-nav and obstacle aversion send control inputs to steering muscles that jointly inhibit wing movement, generating an intermediate response through mechanical interference, and (2) the control requirements are summated neuronally to form a single output signal that is then sent to steering muscles. The answer is to be found in more subtle electrophysiological investigation of the *H. fusca* descending neurons, as in Nicholas et al. (2018). Interference could also be generated at the sensory level. We have assumed that *H. fusca* remains visually fixated on the target owing to the small angular size of the target, yet the obstacle avoidance pathway would also be advantaged by the use of the high-acuity central region of the eye in gauging responses (Tistarelli and Sandini, 1993). Further data, including head tracking data, are required to confirm that *H. fusca* do remain head fixated on the target throughout the trajectory, and to clarify whether they use their central foveal region for obstacle avoidance when a target is not present.

The behaviour displayed by *H. fusca* in their predatory trajectories demonstrates that there is much to learn from insect navigation systems. Layering and weighting control priorities allows *H. fusca* to deal with multiple objectives simply and simultaneously. By summing the different control algorithms, a navigator could add further objectives (e.g. a second obstacle) with relative ease. Combined guidance demonstrates a helpful representation of how optomotor responses toward a moving target and around obstacles can be linearly combined in real time within an animal freely traversing its environment.

## Supplementary Material

Supplementary information
