# Supplementary material for: Avoiding obstacles while intercepting a moving target: a miniature fly's solution
Source: J Exp Biol. 2022 Feb 15;225(4):jeb243568. doi: 10.1242/jeb.243568 (PMC8920034; doi:10.1242/jeb.243568)
Supplement: Supplementary information [file jexbio-225-243568-s1.pdf]

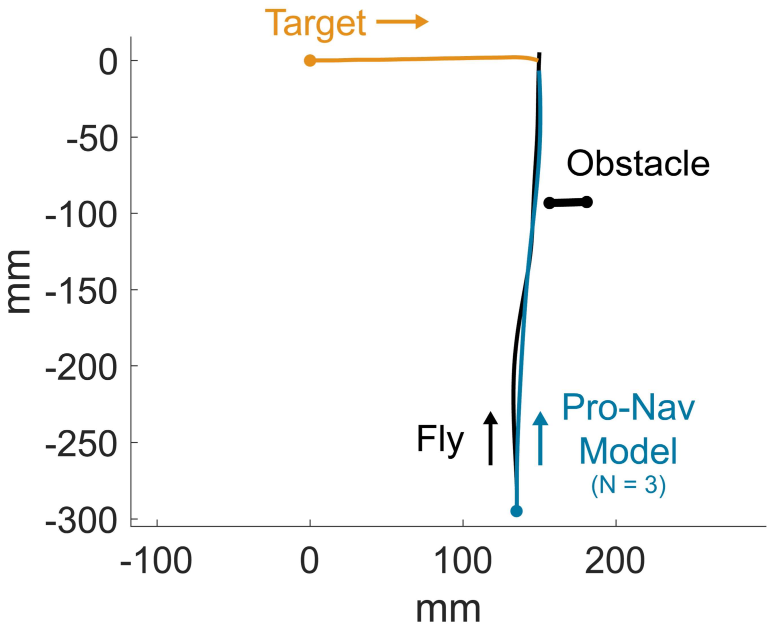

Fig. S1. *Holcocephala* rises to intercept a target in close proximity to a visually salient obstacle, but without demonstrating a path diversion away from it.

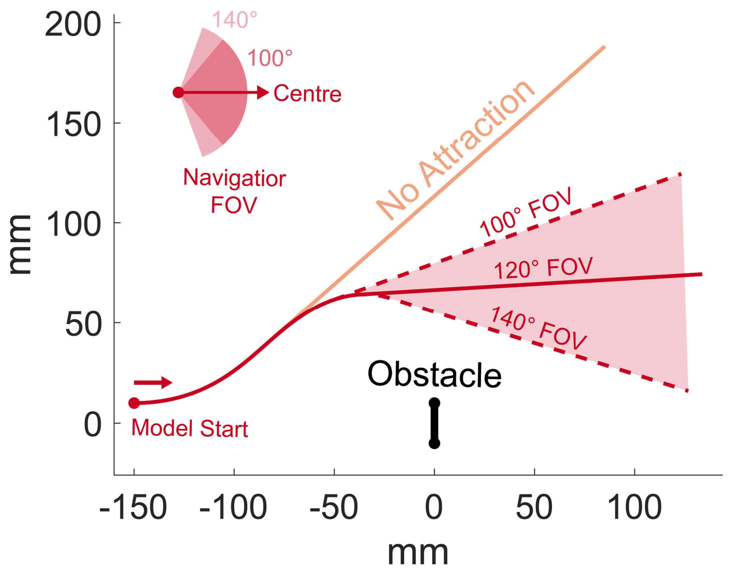

Fig. S2. Simulations travel from left-to-right across the page. The orange trace reacts only to the expansion of the object in the visual field. Red lines are attracted to the visual contraction of the bar as they pass by, so long as it is within their visual field. Attracted simulations are represented with alternative fields (100°, 120° and 140°) of view to demonstrate its effect on the trajectory.

Journal of Experimental Biology • Supplementary information

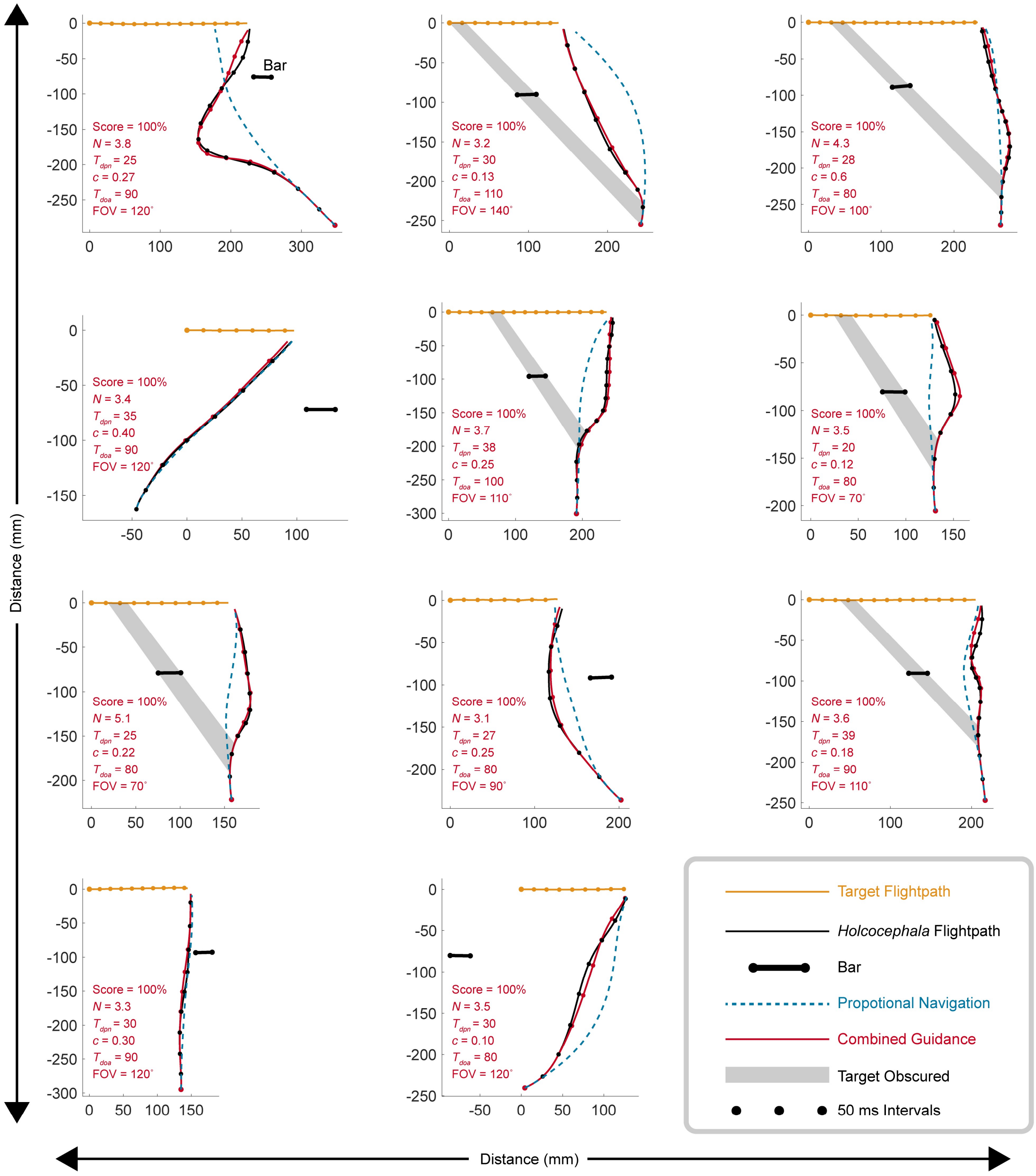

Fig. S3. All completed trajectories in the presence of the thin (2.5 cm) acetate bar are plotted. Overlaid is the best fitting receding attractant combined guidance model (in red, constants detailed in each panel) and a proportional navigation model taken from existing literature (N = 3, T_{dpn} = 28).

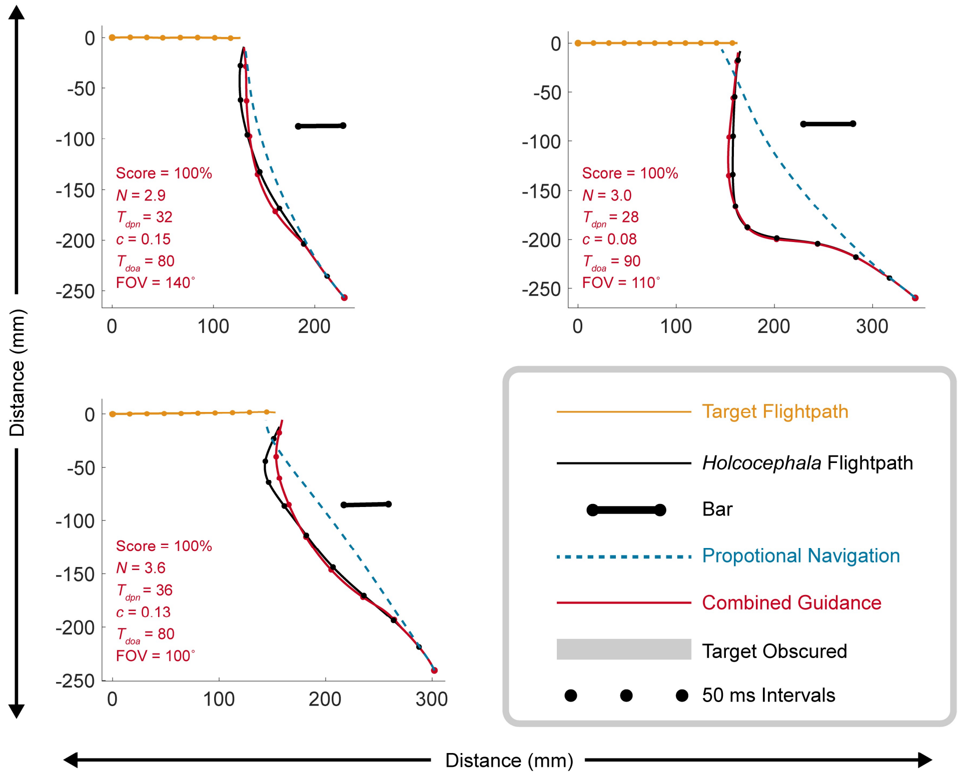

Fig. S4. All completed trajectories in the presence of the thick (5 cm) acetate bar are plotted. Overlaid is the best fitting receding attractant combined guidance model (in red, constants detailed in each panel) and a proportional navigation (in blue) model taken from existing literature (N = 3, T_{dpn} = 28 ms).

Journal of Experimental Biology • Supplementary information

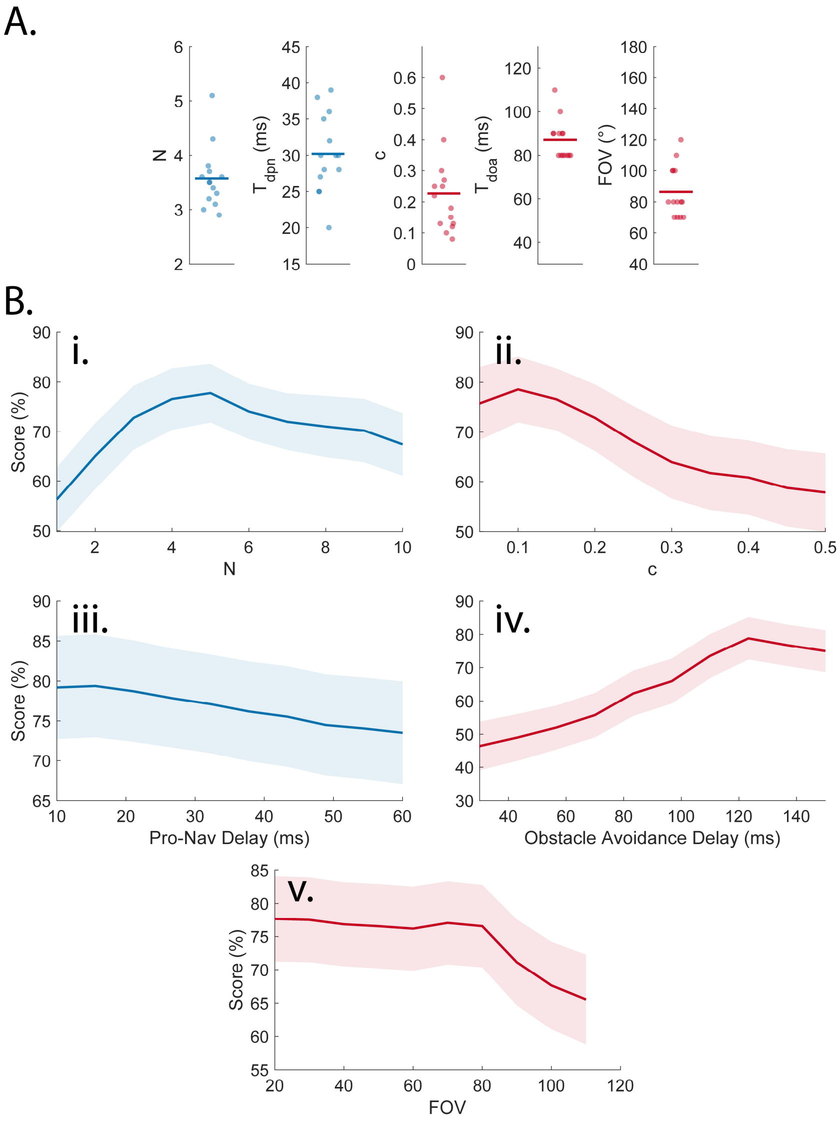

Fig. S5. (A) The best fitting constants for each trajectory are plotted (circles) along with the mean value (Line). **(B)** The sensitivity of the score to variations in the fitted constants is demonstrated by fixing all constants at values close to their best-fitting averages, and varying them each in turn, measuring the mean fit of the model over 14 successful trajectories of *Holcocephala* intercepting a target. The constant values of the base-model were N = 3.5, T_{dpn} = 30 ms, c = 0.23, T_{doa} = 85 ms, FOV = 90°. The constants varied in each panel are: **(i)** Pro-Nav constant N, **(ii)** avoidance constant *c*, **(iii)** pro-nav latency, **(iv)** avoidance latency, **(v)** avoidance field of view. Lines give mean values (n = 14), while shaded regions are +/- standard error.

Journal of Experimental Biology • Supplementary information
